# Pandemic (H1N1) 2009 Infection in Patients with Hematologic Malignancy

**DOI:** 10.3201/eid1612.100772

**Published:** 2010-12

**Authors:** Catherine Liu, Brian S. Schwartz, Snigdha Vallabhaneni, Michael Nixon, Peter V. Chin-Hong, Steven A. Miller, Charles Chiu, Lloyd Damon, W. Lawrence Drew

**Affiliations:** Author affiliation: University of California, San Francisco, California, USA

**Keywords:** Pandemic (H1N1) 2009, viruses, hematologic malignancy, hematopoietic cell transplant, nosocomial transmission, oseltamivir resistance, influenza, research

## MEDSCAPE CME

Medscape, LLC is pleased to provide online continuing medical education (CME) for this journal article, allowing clinicians the opportunity to earn CME credit. This activity has been planned and implemented in accordance with the Essential Areas and policies of the Accreditation Council for Continuing Medical Education through the joint sponsorship of Medscape, LLC and Emerging Infectious Diseases. Medscape, LLC is accredited by the ACCME to provide continuing medical education for physicians. Medscape, LLC designates this educational activity for a maximum of 0.5 *AMA PRA Category 1 Credits*™. Physicians should only claim credit commensurate with the extent of their participation in the activity. All other clinicians completing this activity will be issued a certificate of participation. To participate in this journal CME activity: (1) review the learning objectives and author disclosures; (2) study the education content; (3) take the post-test and/or complete the evaluation at www.medscapecme.com/journal/eid; (4) view/print certificate.

## Learning Objectives

Upon completion of this activity, participants will be able to:

- Distinguish the most common presenting symptom of pandemic (H1N1) 2009 infection in the current study
- Analyze the course of lower respiratory tract infection with pandemic (H1N1) 2009 among patients with hematologic malignancy
- Develop appropriate management strategies for pandemic (H1N1) 2009 infection for patients with hematologic malignancy

## Medscape CME Editor

**Karen L. Foster, MA**, Technical Writer-Editor, *Emerging Infectious Diseases. Disclosure: Karen L. Foster, MA, has disclosed no relevant financial relationships.*

## Medscape CME AUTHOR

**Charles P. Vega, MD**, Associate Professor; Residency Director, Department of Family Medicine, University of California, Irvine. *Disclosure: Charles P. Vega, MD, has disclosed no relevant financial relationships.*

## AUTHORS

*Disclosures:* ***Catherine Liu, MD; Brian S. Schwartz, MD; Snigdha Vallabhaneni, MD; Michael Nixon, NP; Peter V. Chin-Hong, MD Steven A. Miller, MD, PhD;*** *and* ***W. Lawrence Drew, MD, PhD*** *, have disclosed no relevant financial relationships.* ***Charles Chiu, MD, PhD,*** *has disclosed the following relevant financial relationship: served as a speaker or a member of a speakers bureau for Cubist Pharmaceuticals, Inc.* ***Lloyd Damon, MD,*** *has disclosed the following relevant financial relationships: served as a speaker or a member of a speakers bureau for Celgene Corporation; Eisai Inc.; owns stock, stock options, or bonds from Genentech, Inc.*
