# Supplementary material for: Pandemic (H1N1) 2009 Infection in Patients with Hematologic Malignancy
Source: Emerg Infect Dis. 2010 Dec;16(12):1910–7. doi: 10.3201/eid1612.100772 (PMC3294592; doi:10.3201/eid1612.100772)
Supplement: Related texts

# Research article: Pandemic (H1N1) 2009 Infection in Patients with Hematologic Malignancy

**DOI:** 10.3201/eid1612.100772

**Authors:** Catherine Liu, Brian S. Schwartz, Snigdha Vallabhaneni, Michael Nixon, Peter V. Chin-Hong, Steven A. Miller, Charles Chiu, Lloyd Damon, W. Lawrence Drew

**Affiliations:** Author affiliation: University of California, San Francisco, California, USA

## Abstract

To assess outcomes of patients with hematologic malignancy and pandemic (H1N1) 2009 infection, we reviewed cases during June–December 2009 at the University of California San Francisco Medical Center. Seventeen (63%) and 10 (37%) patients had upper respiratory tract infection (URTI) and lower respiratory tract infection (LRTI), respectively. Cough (85%) and fever (70%) were the most common signs; 19% of patients had nausea, vomiting, or diarrhea. Sixty-five percent of URTI patients were outpatients; 35% recovered without antiviral therapy. All LRTI patients were hospitalized; half required intensive care unit admission. Complications included acute respiratory distress syndrome, pneumomediastinum, myocarditis, and development of oseltamivir-resistant virus; 3 patients died. Of the 3 patients with nosocomial pandemic (H1N1) 2009, 2 died. Pandemic (H1N1) 2009 may cause serious illness in patients with hematologic malignancy, primarily those with LRTI. Rigorous infection control, improved techniques for diagnosing respiratory disease, and early antiviral therapy can prevent nosocomial transmission and optimize patient care.

Influenza is a major cause of illness and death in patients with hematologic malignancy and in hematopoietic cell transplant (HCT) recipients. In up to 30% of HCT recipients, illness progressed to lower respiratory tract infection (LRTI); death rates were 28% for patients in whom pneumonia developed (*1*). During spring 2009, infection caused by pandemic (H1N1) 2009 virus emerged in Mexico and spread rapidly throughout the world (*2**–**4*). Although it does not appear to be associated with higher death rates than seasonal influenza (*5*), pandemic (H1N1) 2009 virus has caused severe disease and death, particularly in persons with preexisting illnesses (*6**,**7*). Little is known about the clinical features and outcomes of pandemic (H1N1) 2009 infection in patients with hematologic malignancy. One recently published study suggested that pandemic (H1N1) 2009 causes mild disease in most patients with hematologic malignancy (*8*), but several reports have been published about patients with severe infection and respiratory failure (*9**–**11*). Risk factors for progression to LRTI are unknown. To characterize the clinical spectra and outcomes of pandemic (H1N1) 2009 disease in patients with hematologic malignancy, we reviewed the first 27 cases of pandemic (H1N1) 2009 among these patients in the University of California San Francisco Medical Center during June 1–December 31, 2009.

## Methods

### Patients and Setting

The University of California San Francisco (UCSF) Medical Center (San Francisco, CA, USA) is a large, academic medical center with an active HCT program for children and adults and extensive experience treating children and adults with hematologic malignancy. In 2009, HCTs were performed for 171 adults and 50 children; a total of 1,020 adults and 782 children were admitted to the hospital’s hematology/HCT service.

At UCSF, all nasal swabs performed to diagnose respiratory viral infection in inpatients and outpatients are routinely submitted for testing to the UCSF Virology Laboratory. For infection control surveillance during the pandemic (H1N1) 2009 outbreak, the UCSF Virology Laboratory generated a list of all laboratory-confirmed cases of influenza A during June–December 2009. HCT recipients and other patients with hematologic malignancy were identified through a retrospective chart review of all laboratory-confirmed cases of influenza A during this period. We used a standardized form to capture demographic data, clinical signs and symptoms, underlying hematologic disease and other medical conditions, transplant history, immunosuppressive medications, selected laboratory tests, radiographic findings, treatment course, and clinical outcomes. Dosing and duration of antiviral treatment with oseltamivir or zanamivir, use of concomitant antimicrobial therapy, and intravenous immunoglobulin was determined by the treating providers. The study protocol was approved by the UCSF Committee on Human Research.

### Laboratory Confirmation of Infection

All diagnostic testing, including repeat serial testing, was performed at the discretion of the treating provider. The standard clinical practice for detecting respiratory viral infection, including influenza, was to obtain a nasopharyngeal wash, aspirate, or flocked swab for viral direct fluorescent antibody (DFA) testing (D^3^-DFA Respiratory Virus Screening and ID Kit, Diagnostics Hybrids, Athens, OH, USA) with same-day turnaround. Specimens from high-risk immunocompromised patients were submitted for multiplex PCR testing (xTAG RVP [Respiratory Viral Panel]; Luminex, Austin, TX, USA) if DFA results were negative. Results were considered consistent with pandemic (H1N1) 2009 infection when specimens were positive for influenza A by DFA or positive for influenza A matrix gene but negative for H1 and H3 hemagglutin gene subtypes by RVP. Pandemic (H1N1) 2009 infection was confirmed by using banked frozen specimens with at least 1 of 3 PCRs; the Xpert Flu A Panel (Cepheid Corp, Sunnyvale, CA, USA), the Centers for Disease Control and Prevention (CDC) real-time reverse transcription–PCR (rRT-PCR) swine influenza panel (performed at the San Francisco Department of Public Health), or a previously described PCR specific for pandemic (H1N1) 2009 performed in our laboratory (*12*). Patients were considered to have probable pandemic (H1N1) 2009 if laboratory-confirmed influenza A was detected during June–December 2009 and additional specimens were not available for confirmatory testing. CDC performed pyrosequencing to detect the H275Y mutation in the N1 neuraminidase gene associated with oseltamivir resistance.

### Definitions

Upper respiratory tract infection (URTI) was defined as pandemic (H1N1) 2009 virus in nasopharyngeal specimen and compatible clinical symptoms without new pulmonary infiltrates on chest radiograph. Lower respiratory tract infection (LRTI) was defined as pandemic (H1N1) 2009 virus in a nasopharyngeal, endotracheal tube, or bronchoalveolar lavage specimen and compatible clinical symptoms with a new pulmonary infiltrate on chest radiograph or computed tomography (CT) imaging.

### Statistical Analysis

We compared patient median age, absolute lymphocyte count, and duration of antiviral therapy by using the Wilcoxin rank-sum test. For categorical variables, we calculated the proportions of patients in each category. Clinical characteristics, therapy, and outcomes were compared between subgroups of patients by using the Fisher exact test. We identified independent predictors of LRTI using logistic regression. Predictors significant at p<0.10 in both the univariate and multivariate analyses were retained in the final multivariate models. We conducted analyses using Stata software version 9.0 (StataCorp, College Station, TX, USA).

## Results

During June 1–December 31, 2009, a total of 159 probable or laboratory-confirmed cases of pandemic (H1N1) 2009 infection were identified at our institution. Eighteen (11%) were HCT recipients and 9 (6%) had a hematologic malignancy and were included in the study. The number of identified cases peaked early in the pandemic in June, although ≈40% of the hematologic malignancy patients sought care for influenza in November (Figure 1).

**Figure 1:** Pandemic (H1N1) 2009 cases among hematologic malignancy patients compared with all other patients, University of California San Francisco Medical Center, San Francisco, California, USA, June–December 2009.

Median age of patients was 43 years, and patients with URTI were younger than those with LRTI (33 vs. 53 years; p = 0.04) (Table 1). Two patients were children; both had URTI. Two thirds of cases occurred in male patients. Among HCT recipients, the median time of symptom onset after transplant was 12 months (range 0.3–45 months). A total of 10 (37%) patients had LRTI; compared with patients with URTI, these patients were older, significantly more likely to have diabetes mellitus or underlying lung disease, and more likely to be receiving corticosteroids. After controlling for sex, transplant status, presence of chronic lung disease, and corticosteroid use, we found that older age was independently associated with development of LRTI (odds ratio [OR] 1.15; 95% confidence interval [CI] 1.02–1.30; p = 0.0001).

**Table 1:** Characteristics of hematologic malignancy patients with URTI or LRTI and pandemic (H1N1) 2009 virus, University of California, San Francisco, Medical Center, San Francisco, California, USA, June–December 2009*

Characteristic	Total, n = 27	URTI, n = 17	LRTI, n = 10	p value†
Median age, y (range)	43 (5–83)	33 (5–83)	53 (29–80)	0.04
Male sex	18 (67)	10 (59)	8 (80)	0.24
Underlying malignancy				
Acute lymphocytic leukemia	6 (22)	5 (29)	1 (10)	0.25
Acute myelocytic leukemia	5 (19)	4 (24)	1 (10)	0.37
Chronic lymphocytic leukemia	1 (4)	1 (6)	0	0.63
Lymphoma	6 (22)	2 (12)	4 (40)	0.11
Multiple myeloma	7 (26)	4 (24)	3 (30)	0.52
Other	2 (7)	1 (6)	1 (10)	0.61
Hematopoietic cell transplant	18 (67)	12 (71)	6 (60)	0.44
Allogeneic	7 (39)	4 (33)	3 (50)	0.43
Graft-vs.-host disease	6 (86)	3 (75)	3 (100)	0.57
Autologous	11 (61)	8 (67)	3 (50)	0.43
Median time posttransplant, mo (range)	12 (0.3–45)	12 (3–45)	11 (0.3–34)	0.81
Immunosuppressive medications				
Corticosteroid use	7 (26)	2 (12)	5 (50)	0.04
T-/B-cell depleting agent	4 (15)	1 (6)	3 (30)	0.13
Underlying concurrent conditions				
Obesity, body mass index >30 kg/m^2^	2 (7)	0	2 (20)	0.13
Chronic lung disease	5 (19)	1 (6)	4 (40)	0.047
Diabetes mellitus	6 (22)	1 (6)	5 (50)	0.015
HIV infection	2 (7)	1 (6)	1(10)	0.61

*Values are given as no. (%) patients except as indicated. URTI, upper respiratory tract infection; LRTI, lower respiratory tract infection. †p value represents statistical difference between comparison of values of patients with LRTI and URTI.

Cough (85%) and fever (70%) were the most common signs; 5 (19%) patients had nausea, vomiting, or diarrhea (Table 2). Compared with patients with URTI, those with LRTI were more likely to have dyspnea (12% vs. 90%; p<0.01) and less likely to have rhinorrhea (65% vs. 10%; p = 0.005) and had a lower median absolute lymphocyte count (ALC) (815 cells/μL vs. 130 cells/μL; p = 0.02). All 10 patients with LRTI were hospitalized, compared with 35% of patients with URTI (p = 0.001). Five (50%) LRTI patients required monitoring in the intensive care unit (ICU), of whom 4 required mechanical ventilation because of respiratory failure. Pulmonary infiltrates observed on CT scan of the LRTI patients ranged from focal consolidations (7/10) to ground glass opacities (6/10), centrilobular nodules (2/10), and tree-in-bud opacities (2/10).

**Table 2:** Clinical features, treatments, and outcomes for hematologic malignancy patients who had URTI and LRTI from pandemic (H1N1) 2009 virus, University of California San Francisco Medical Center, San Francisco, California, USA, June–December 2009*

Characteristic	Total, n = 27	URTI, n = 17	LRTI, n = 10	p value†
Signs and symptoms				
Fever	19 (70)	11 (65)	8 (80)	0.44
Cough	23 (85)	14 (82)	9 (90)	0.68
Shortness of breath	11 (41)	2 (12)	9 (90)	<0.01
Myalgias	4 (15)	3 (18)	1 (10)	0.50
Rhinorrhea	12 (44)	11 (65)	1 (10)	0.005
Sore throat	8 (30)	6 (35)	2 (20)	0.31
Gastrointestinal symptoms‡	5 (19)	3 (18)	2 (20)	0.66
Household influenza exposure§	8 (30)	1 (6)	7 (70)	0.001
Laboratory values				
ALC, cells/μL, median (range)	570 (0–16,370)	815 (150–16,370)	130 (0–1,860)¶	0.02
Absolute neutrophil count <500 cells/μL	5 (19)	3 (19)	2 (20)	0.66
Treatment#				
Antiviral drug therapy	21 (78)	11 (65)	10 (100)	0.042
Symptom onset to start of antiviral drug therapy, h**				
<48	4 (19)	3 (27)	1 (10)	0.29
48–96	5 (24)	2 (18)	3 (30)	0.50
>96	11 (52)	5 (45)	6 (60)	0.50
Type of antiviral drug therapy				
Oseltamivir, standard dose	8 (38)	5 (45)	3 (30)	0.65
Oseltamivir, high dose††	11 (52)	4 (36)	7 (70)	0.02
Zanamivir, inhaled	2 (10)	0	2 (20)	0.13
Zanamivir, intravenous	1 (5)	0	1 (10)	0.37
Median duration of antiviral drug therapy, d (range)	7 (5–49)	5 (5–14)	15 (5–49)	0.058
Intravenous immunoglobulin	6 (22)	1 (9)	5 (50)	0.015
Outcome				
Hospitalization	16 (59)	6 (35)	10 (100)	0.001
Intensive care unit admission	5 (19)	0	5 (50)	0.003
Mechanical ventilation	4 (15)	0	4 (40)	0.012
Death	3 (11)	0	3 (30)	0.041

*Values are given as no. (%) patients except as indicated. URTI, upper respiratory tract infection; LRTI, lower respiratory tract infection; ALC, absolute leukocyte count. †p value represents statistical difference between comparison of values of patients with LRTI and URTI. ‡Nausea, vomiting, and/or diarrhea. §Exposure to an ill household contact documented in patient’s chart. ¶Two LRTI patients had ALC below the level of detection, and the value was reported by the laboratory as <100 lymphocytes/μL. For this calculation, these patients were assigned an ALC value of 0. #The calculation for LRTI excludes 2 patients who received only 2–3 d of antiviral therapy just before they died because their anticipated duration of antiviral therapy would have been longer. **Time of symptom was available for 20 of 21 patients who received antiviral therapy. ††Two patients received standard-dose and high-dose oseltamivir.

Influenza A was diagnosed for 21 (78%) patients by DFA. DFA results were negative for 6 (22%) patients, but influenza A was subsequently diagnosed among them by PCR. Isolates for all patients were confirmed as pandemic (H1N1) 2009 virus by PCR, except for 2 for whom additional specimens were not available for confirmatory testing. These were considered probable cases of pandemic (H1N1) 2009 because no cases of seasonal H1 or H3 influenza were laboratory confirmed during this time.

Twenty-one (78%) patients received antiviral therapy, and 20 (95%) received standard-dose or high-dose oseltamivir. Two patients, both with LRTI, received inhaled zanamivir; intravenous zanamivir was later used for 1 of these patients who required mechanical ventilation and had oseltamivir-resistant virus (patient 3 in Figure 2; Table 3). Four (19%) patients received antiviral therapy within 48 hours after symptom onset; treatment started >96 hours after symptom onset for more than half the patients. Median duration of antiviral therapy was 5 days (range 5–14 days) and 15 days (range 5–49 days) for patients with URTI and LRTI, respectively. No concomitant bacterial infections were identified; however, 20 (74%) patients were empirically prescribed broad-spectrum antibacterial therapy. Invasive pulmonary *Aspergillus fumigatus* was concurrently diagnosed in 1 patient. Six (35%) patients with URTI were not treated with antiviral therapy and recovered without sequelae.

**Figure 2:** Clinical course for a 45-year-old woman (Table 3) hospitalized (periods indicated by gray shading) with influenza-associated pneumonia and concurrent pulmonary aspergillosis. The patient had received an autologous stem cell transplant 1 year earlier and underwent treatment with high-dose (HD) steroids for carmustine (BCNU) pneumonitis. On admission, she received HD oseltamivir (150 mg orally 2×/d) for 14 days, and antifungal therapy was initiated. Test results were positive for wild-type (WT) pandemic (H1N1) 2009 by PCR and influenza A by direct fluorescent antibody (DFA). A repeat pandemic (H1N1) 2009 PCR result was positive 1 week into treatment, but her condition later improved, and she was discharged to home. Ten days later, she was readmitted to the intensive care unit (ICU) with worsening dyspnea and again had positive test results for pandemic (H1N1) 2009; a pulmonary embolus was found. HD oseltamivir was restarted, but when pandemic (H1N1) 2009 PCR results remained persistently positive, she was switched to inhaled (INH) zanamivir and then intravenous (IV) zanamivir after intubation. PCR results indicated the H275Y mutation, confirming oseltamivir resistance. She eventually showed evidence of viral clearance but died of respiratory failure secondary to adult respiratory distress syndrome, pulmonary embolus, progressive pulmonary aspergillosis, and BCNU pneumonitis. PCR results were returned 5–11 days after specimen submission.

**Table 3:** Selected cases of severe pandemic (H1N1) 2009 infection in patients with hematologic malignancy, University of California San Francisco Medical Center, San Francisco, California, USA, June–December 2009*

Patient no./ age, y/sex	Underlying disease, time frame	ALC†	Neutro	Onset of symptoms, d before antiviral therapy	Antiviral therapy; duration, d	Duration of viral shedding, d‡	Hospital acquired	ICU/ mechanical ventilation, d	Complications; outcome
1/43/M	APML, 9 d post auto-SCT	<100†	Yes	6	SD/HD oseltamivir;§ 40 d	26	Yes	25/8	Myocarditis, cardiogenic shock; survived
2/48/M	HIV, Burkitt lymphoma	<100†	Yes	7	HD oseltamivir; 2 d	Unknown	Yes	5/4	ARDS; died
3/45/F	DLBCL, 1 year post auto-SCT, BCNU pneumonitis	80	No	5	HD oseltamivir, inhaled/IV zanamivir; 49 d	48	No	40/13	Oseltamivir resistance; died
4/75/M	Multiple myeloma	40	No	7	HD oseltamivir; 2 d	Unknown	Yes	4/3	ARDS, died
5/29/M	ALL, 1-m post allo-SCT	160	No	3	HD oseltamivir; 20 d	7	No	8/0	Pneumomediastinum; pneumopericardium, bronchiolitis obliterans; survived

*ALC, absolute lymphocyte count/μL; Neutro, neutropenia; ICU, intensive care unit; APML, acute promyelocytic leukemia; SCT, stem cell transplant; SD, standard dose; HD, high dose; DLBCL, diffuse large B-cell lymphoma; IV, intravenous; BCNU, carmustine. ARDS, adult respiratory distress syndrome; ALL, acute lymphocytic leukemia. †Two patients had an ALC below the level of detection, and the laboratory reported the value as <100 cells/μL. ‡This time is the minimum estimated duration of viral shedding calculated on the basis of the time between the first positive to the last positive specimen collected. Because there was no standard collection interval between specimens and specimens were not collected >1 time weekly, viral shedding may have been longer than indicated. For example, patient 5 had 2 positive specimens collected 7 d apart; his next specimen, collected 15 d later, was negative. §Oseltamivir dosing varied from HD at 150 mg 2×/d to SD at 75 mg 2×/d.

Five (19%) of 27 patients experienced severe complications related to pandemic (H1N1) 2009 infection that required admission to ICU, and 3 (11%) died (Table 3). Of these patients with severe infection, 3 (patients 1, 2, and 4) acquired the infection during hospitalization. None had clinical evidence of a respiratory viral infection at admission. Retrospective chart review suggested that symptoms began at 13, 16, and 11 days after admission, respectively. For all 3 patients, new onset of fever (2 with neutropenia), mild cough, progressive dyspnea, and hypoxia developed; however, the latter did not become prominent in 2 patients until 4–6 days after initial onset of symptoms. Two patients had diarrhea. Two of the 3 had ground glass opacities with areas of focal consolidation on chest CT scan; the third had frank multilobar consolidation. For each patient, initial evaluation focused on workup for typical hospital-acquired bacterial and opportunistic infections; a respiratory viral infection was not considered until 4–7 days after symptom onset, which resulted in a delay in diagnostic testing and initiation of antiviral therapy of ≈1 week. For 2 patients (2 and 4), DFA results were negative, and diagnosis was ultimately made by PCR; the result was returned postmortem for patient 4. Pandemic (H1N1) 2009 infection resulted in the deaths of 2 of these 3 patients; the third (patient 1) had substantially delayed engraftment requiring an infusion of back-up autologous stem cells; myocarditis with cardiogenic shock developed, but the patient eventually recovered. None of these cases were clustered, and the source of infection was not clearly identified but was presumed to be an ill healthcare worker (HCW) or visitor.

The third fatal case occurred in a patient who had received an autologous stem cell transplant 1 year before illness that was complicated by carmustine pneumonitis requiring steroid therapy (patient 3; Figure 2). She was initially admitted with wild-type pandemic (H1N1) 2009 infection and concurrent pulmonary aspergillosis; she improved after completing 14 days of oseltamivir therapy and initiation of antifungal therapy. Ten days after hospital discharge, she was readmitted with recurrent dyspnea and had persistent viral shedding with what was later confirmed as oseltamivir-resistant pandemic (H1N1) 2009 virus and a new pulmonary embolus. She received inhaled, and then intravenous, zanamivir and demonstrated evidence of viral clearance. However, in the context of her adult respiratory distress syndrome (ARDS), pulmonary embolus, progressive pulmonary aspergillosis, and carmustine pneumonitis, she ultimately died of respiratory failure.

## Discussion

We describe a consecutive series of 27 hematologic malignancy patients with pandemic (H1N1) 2009 infection, including 3 nosocomial cases, during June–December 2009. The spectrum of illness severity ranged from mild to severe, and most patients in this and other series had signs and symptoms suggestive of an influenza-like illness (*8*). Similar to reported signs of pandemic (H1N1) 2009 in immunocompetent hosts (*13**,**14*), cough and fever were the most common signs, and nausea, vomiting, and diarrhea occurred in a greater proportion than typically observed for seasonal influenza (*13**–**15*). Although 17 (63%) patients had URTI, with 11 (65%) managed as outpatients, all 10 (37%) patients with LRTI required hospitalization. Risk factors for LRTI included chronic lung disease, diabetes mellitus, and more marked immunosuppression as measured by corticosteroid use and lymphopenia at diagnosis. Although previous observations suggest that older age protects against pandemic (H1N1) 2009 infection (*7**,**16**,**17*), after infection is established in persons with hematologic malignancy, older age predicted an increased risk for disease involving the lower respiratory tract. These findings may be due to the lack of preexisting antibodies, which could have conferred partial immunity, in patients with hematologic malignancy.

Half of the patients with LRTI required ICU admission, and all except 1 needed mechanical ventilation. In addition to ARDS, several pandemic (H1N1) 2009–related complications were observed. One patient developed spontaneous pneumomediastinum, thought to be secondary to viral bronchiolitis from pandemic (H1N1) 2009. Spontaneous pneumomediastinum has been reported as a complication of influenza, including pandemic (H1N1) 2009, in adults and children (*18**,**19*). In another patient, severe myocarditis associated with cardiogenic shock developed; the patient ultimately had partial recovery of his left ventricular ejection fraction with treatment and supportive care. Three of the 10 patients with LRTI died, similar to published death rates on seasonal influenza pneumonia in HCT recipients (*1*).

Because decisions about antiviral therapy were made by different providers, the dose, duration, and timing of antiviral therapy relative to symptom onset varied substantially. Most patients with URTI appeared to respond to 5 days of therapy, but about one third recovered without antiviral therapy. Most patients with LRTI received high-dose oseltamivir during their treatment course, but whether this dosage is more effective than standard-dose oseltamivir is not possible to conclude. Because only 19% of patients began antiviral therapy within 48 hours after symptom onset, assessing the effect of early antiviral therapy was difficult. However, none of the 5 patients requiring ICU admission received antiviral therapy within 48 hours after symptom onset. Although the vaccination status of all patients is unknown, pandemic (H1N1) 2009 vaccine was not available during most of the study period.

Shedding of seasonal influenza virus by otherwise healthy adults is ≈5–7 days but can be >1 week for hospitalized patients (*20*) and an average of 11–12 days for HCT recipients (*1**,**21*). Initiation of therapy within the first 4 days of illness appears to enhance viral clearance (*20*). Two patients had laboratory evidence of viral shedding beyond 2 weeks; for both, antiviral therapy was started >5 days after symptom onset. Testing for the H275Y mutation showed that oseltamivir-resistant virus developed in 1 person after 14 days of therapy. This patient had persistent viral shedding after a week of therapy, and testing was not repeated before cessation of therapy; whether ongoing viral shedding may have selected for development of oseltamivir-resistant virus is unclear. Consistent with recommendations made by Casper et al. (*22*), we believe that hematologic malignancy patients with LRTI should be treated with high-dose oseltamivir for a minimum of 10 days. We recommend weekly monitoring of such patients with serial viral PCR testing and extended antiviral therapy until PCR is negative, particularly for patients who have ongoing clinical symptoms or are severely immunocompromised.

Three cases of oseltamivir-resistant pandemic (H1N1) 2009 in patients with HCT have been published. These patients shed virus for 6–8 weeks, and oseltamivir-resistant virus developed after 5, 11, and 21 days of oseltamivir therapy (*23**,**24*). It was difficult to determine the relative contribution of oseltamivir-resistant pandemic (H1N1) 2009 to the overall clinical course and ultimate death of the case-patient in our report, given her ARDS and multiple concurrent pulmonary disease processes. Asymptomatic viral shedding has been described for infections with other respiratory viruses, such as parainfluenza and respiratory syncytial virus (*25**,**26*), and the recovery of 1 patient with oseltamivir-resistant virus without zanamivir therapy is notable (*23*). Further research is needed to determine the clinical significance of persistent viral shedding, role of antiviral therapy in this situation, and risk factors for oseltamivir resistance in this patient population.

Nosocomial transmission of pandemic (H1N1) 2009 to 3 patients resulted in serious complications and prolonged hospitalization of 1 patient and deaths of 2 patients. Although these cases occurred independently, ascertaining the source of transmission (HCW vs. visitor) was difficult. For each patient, influenza was not initially suspected, resulting in delayed diagnostic testing and initiation of antiviral therapy. For 2 patients, diagnosis was further delayed by initial negative DFA results and positive PCR returning 5–10 days later. Performance characteristics of the DFA test for detecting pandemic (H1N1) 2009 have been shown to vary from a sensitivity of 47% and negative predictive value of 59% (*27*) to a sensitivity of 93% and negative predictive value of 96% (*28*). In our case series, 22% of patients had negative DFA results and subsequent positive results for pandemic (H1N1) 2009 by PCR. Rapid, highly sensitive, and specific tests clearly are needed for detecting influenza, including pandemic (H1N1) 2009, in combination with rigorous infection control strategies. In response to these in-hospital transmissions, multiple measures were implemented on the hematology and blood and marrow transplant unit to prevent further transmission, including 1) visitor screening, with required symptom review, before allowing entry into patient rooms; 2) restriction to 2 visitors at any given time in patient room; 3) reinforcement of “stay at home when sick” policy among HCWs (we relied on an honor system and asked ill HCWs to call in sick rather than come in to be screened); 4) closure of common pantry area on the unit to patients and visitors; and 5) maintenance of droplet precautions for patients with pandemic (H1N1) 2009 infection throughout their hospitalization. Several hospitalwide measures were implemented: 1) exclusion of all visitors <16 years of age; 2) a policy of mandatory seasonal and pandemic (H1N1) 2009 vaccination for all HCWs or a requirement for those who declined to wear masks in patient-care areas; 3) reinforcement of “stay at home when sick” policy among HCWs; 4) intensive education about hand hygiene, use of appropriate precautions emphasizing early isolation of patients with influenza-like illness; and 5) proposed acquisition of in-house rapid PCR testing system for influenza.

Our findings indicate that investing in the development of enhanced diagnostic methods for respiratory disease is critical to ensure timely and accurate diagnoses. In addition, further research is needed to define the optimal dose, duration, and choice of antiviral therapy for managing influenza infections in immunocompromised patients. Finally, aggressive infection control measures are crucial for preventing transmission of pandemic (H1N1) 2009 and other respiratory viral diseases in patients with hematologic malignancy.

## References

[1] Nichols WG, Guthrie KA, Corey L, Boeckh M. Influenza infections after hematopoietic stem cell transplantation: risk factors, mortality, and the effect of antiviral therapy. Clin Infect Dis. 2004;39:1300–6. 10.1086/42500415494906

[2] Centers for Disease Control and Prevention. Swine influenza A (H1N1) infection in two children—southern California, March–April 2009. MMWR Morb Mortal Wkly Rep. 2009;58:400–2.19390508

[3] Centers for Disease Control and Prevention. Outbreak of swine-origin influenza A (H1N1) virus infection—Mexico, March–April 2009. MMWR Morb Mortal Wkly Rep. 2009;58:467–70.19444150

[4] Centers for Disease Control and Prevention. Update: infections with a swine-origin influenza A (H1N1) virus—United States and other countries, April 28, 2009. MMWR Morb Mortal Wkly Rep. 2009;58:431–3.19407737

[5] Presanis AM, de Angelis D, Hagy A, Reed C, Riley S, Cooper BS, The severity of pandemic H1N1 influenza in the United States, from April to July 2009: a Bayesian analysis. PLoS Med. 2009;6:e1000207. 10.1371/journal.pmed.100020719997612PMC2784967

[6] Centers for Disease Control and Prevention. 2009 Pandemic influenza A (H1N1) virus infections—Chicago, Illinois, April–July 2009. MMWR Morb Mortal Wkly Rep. 2009;58:913–8.19713879

[7] Jain S, Kamimoto L, Bramley AM, Schmitz AM, Benoit SR, Louie J, Hospitalized patients with 2009 H1N1 influenza in the United States, April–June 2009. N Engl J Med. 2009;361:1935–44. 10.1056/NEJMoa090669519815859

[8] Redelman-Sidi G, Sepkowitz KA, Huang CK, Park S, Stiles J, Eagan J, 2009 H1N1 influenza infection in cancer patients and hematopoietic stem cell transplant recipients. J Infect. 2010;60:257–63. 10.1016/j.jinf.2010.01.00920138188

[9] Kharfan-Dabaja MA, Velez A, Richards K, Greene JN, Field T, Sandin R. Influenza A/pandemic 2009/H1N1 in the setting of allogeneic hematopoietic cell transplantation: a potentially catastrophic problem in a vulnerable population. Int J Hematol. 2010;91:124–7. 10.1007/s12185-009-0464-520013322PMC7101598

[10] Patel P, Sweiss K, Shatavi S, Peace D, Clark N, Rondelli D. The impact of novel influenza A (H1N1) after hematopoietic SCT. [Epub ahead of print]. Bone Marrow Transplant. 2010;:45.2022885110.1038/bmt.2010.57

[11] Rozovski U, Herishanu Y, Gipstein L, Naparstek E. Fatal H1N1 influenza infection in an allo-SCT recipient. [Epub ahead of print]. Bone Marrow Transplant. 2010;:45.2009845710.1038/bmt.2009.376PMC7091923

[12] Elicker BM, Schwartz BS, Liu C, Chen EC, Miller SA, Chiu CY, Thoracic CT findings of novel influenza A (H1N1) infection in immunocompromised patients. Emerg Radiol. 2010;17:299–307. 10.1007/s10140-010-0859-x20111882PMC2880241

[13] Cao B, Li XW, Mao Y, Wang J, Lu HZ, Chen YS, Clinical features of the initial cases of 2009 pandemic influenza A (H1N1) virus infection in China. N Engl J Med. 2009;361:2507–17. 10.1056/NEJMoa090661220007555

[14] Novel Swine-Origin Influenza A (H1N1) Virus Investigation Team, Dawood FS, Jain S, Finelli L, Shaw MW, Lindstrom S, . Emergence of a novel swine-origin influenza A (H1N1) virus in humans. N Engl J Med. 2009;360:2605–15. 10.1056/NEJMoa090381019423869

[15] Perez-Padilla R, de la Rosa-Zamboni D, Ponce de Leon S, Hernandez M, Quinones-Falconi F, Bautista E, Pneumonia and respiratory failure from swine-origin influenza A (H1N1) in Mexico. N Engl J Med. 2009;361:680–9. 10.1056/NEJMoa090425219564631

[16] Kelly H, Grant K. Interim analysis of pandemic influenza (H1N1) 2009 in Australia: surveillance trends, age of infection and effectiveness of seasonal vaccination. Euro Surveill. 2009;14:pii:19288.10.2807/ese.14.31.19288-en19660248

[17] Miller E, Hoschler K, Hardelid P, Stanford E, Andrews N, Zambon M. Incidence of 2009 pandemic influenza A H1N1 infection in England: a cross-sectional serological study. Lancet. 2010;375:1100–8. 10.1016/S0140-6736(09)62126-720096450

[18] Hasegawa M, Hashimoto K, Morozumi M, Ubukata K, Takahashi T, Inamo Y. Spontaneous pneumomediastinum complicating pneumonia in children infected with the 2009 pandemic influenza A (H1N1) virus. Clin Microbiol Infect. 2010;16:195–9. 10.1111/j.1469-0691.2009.03086.x19832702

[19] Tutor JD, Montgomery VL, Eid NS. A case of influenza virus bronchiolitis complicated by pneumomediastinum and subcutaneous emphysema. Pediatr Pulmonol. 1995;19:393–5. 10.1002/ppul.19501906147567221

[20] Lee N, Chan PK, Hui DS, Rainer TH, Wong E, Choi KW, Viral loads and duration of viral shedding in adult patients hospitalized with influenza. J Infect Dis. 2009;200:492–500. 10.1086/60038319591575PMC7110250

[21] Khanna N, Steffen I, Studt JD, Schreiber A, Lehmann T, Weisser M, Outcome of influenza infections in outpatients after allogeneic hematopoietic stem cell transplantation. Transpl Infect Dis. 2009;11:100–5. 10.1111/j.1399-3062.2008.00362.x19175540

[22] Casper C, Englund J, Boeckh M. How I treat influenza in patients with hematologic malignancies. Blood. 2010;115:1331–42. 10.1182/blood-2009-11-25545520009037PMC2826758

[23] Centers for Disease Control and Prevention. Oseltamivir-resistant novel influenza A (H1N1) virus infection in two immunosuppressed patients—Seattle, Washington, 2009. MMWR Morb Mortal Wkly Rep. 2009;58:893–6.19696719

[24] Gaur AH, Bagga B, Barman S, Hayden R, Lamptey A, Hoffman JM, Intravenous zanamivir for oseltamivir-resistant 2009 H1N1 influenza. N Engl J Med. 2010;362:88–9. 10.1056/NEJMc091089320032317

[25] Anaissie EJ, Mahfouz TH, Aslan T, Pouli A, Desikan R, Fassas A, The natural history of respiratory syncytial virus infection in cancer and transplant patients: implications for management. Blood. 2004;103:1611–7. 10.1182/blood-2003-05-142514525792

[26] Peck AJ, Englund JA, Kuypers J, Guthrie KA, Corey L, Morrow R, Respiratory virus infection among hematopoietic cell transplant recipients: evidence for asymptomatic parainfluenza virus infection. Blood. 2007;110:1681–8. 10.1182/blood-2006-12-06034317502457PMC1975849

[27] Ginocchio CC, Zhang F, Manji R, Arora S, Bornfreund M, Falk L, Evaluation of multiple test methods for the detection of the novel 2009 influenza A (H1N1) during the New York City outbreak. J Clin Virol. 2009;45:191–5. 10.1016/j.jcv.2009.06.00519540158PMC7172175

[28] Pollock NR, Duong S, Cheng A, Han LL, Smole S, Kirby JE. Ruling out novel H1N1 influenza virus infection with direct fluorescent antigen testing. Clin Infect Dis. 2009;49:e66–8. 10.1086/64450219681710PMC2768282

# Research article: Pandemic (H1N1) 2009 Infection in Patients with Hematologic Malignancy

**DOI:** 10.3201/eid1612.100772

**Authors:** Catherine Liu, Brian S. Schwartz, Snigdha Vallabhaneni, Michael Nixon, Peter V. Chin-Hong, Steven A. Miller, Charles Chiu, Lloyd Damon, W. Lawrence Drew

**Affiliations:** Author affiliation: University of California, San Francisco, California, USA

## Earning CME Credit

To obtain credit, you should first read the journal article. After reading the article, you should be able to answer the following, related, multiple-choice questions. To complete the questions and earn continuing medical education (CME) credit, please go to **www.medscapecme.com/journal/eid**. Credit cannot be obtained for tests completed on paper, although you may use the worksheet below to keep a record of your answers. You must be a registered user on Medscape.com. If you are not registered on Medscape.com, please click on the New Users: Free Registration link on the left hand side of the website to register. Only one answer is correct for each question. Once you successfully answer all post-test questions you will be able to view and/or print your certificate. For questions regarding the content of this activity, contact the accredited provider, CME@medscape.net. For technical assistance, contact CME@webmd.net. American Medical Association’s Physician’s Recognition Award (AMA PRA) credits are accepted in the US as evidence of participation in CME activities. For further information on this award, please refer to http://www.ama-assn.org/ama/pub/category/2922.html. The AMA has determined that physicians not licensed in the US who participate in this CME activity are eligible for *AMA PRA Category 1 Credits*™. Through agreements that the AMA has made with agencies in some countries, AMA PRA credit is acceptable as evidence of participation in CME activities. If you are not licensed in the US and want to obtain an AMA PRA CME credit, please complete the questions online, print the certificate and present it to your national medical association.

## Article Title: Pandemic (H1N1) 2009 Infection in Patients with Hematologic Malignancy

## CME Questions

**1. A 43-year-old woman who recently began induction chemotherapy for acute myelocytic leukemia presents with a 3-day history of worsening symptoms, and is concerned about possible influenza. Which of the following symptoms would be most indicative of H1N1 influenza infection?**A. Nausea and vomitingB. Cough and feverC. DiarrheaD. Dyspnea**2. You suspect influenza infection, and perform a nasopharyngeal wash for analysis. You consider initiating antiviral therapy.Which of the following statements about antiviral therapy is most accurate?**A. Most patients were treated within 48 hours of the onset of symptomsB. Many patients received broad-spectrum antibiotics, but no concomitant bacterial infections were foundC. No patients recovered from H1N1 infection without antiviral therapyD. High-dose oseltamivir was more effective than standard-dose therapy**3. The patient is diagnosed with lower respiratory tract infection (LRTI) and admitted into the hospital. Which of the following statements about patients with LRTIs is most accurate?**A. All patients with LRTIs were hospitalizedB. No patients with LRTIs were admitted to the intensive care unitC. Only focal consolidations were seen on chest CT scans of LRTI patientsD. Patient age had no effect on the risk for LRTIs**4. Which of the following treatments is appropriate for this patient?**A. Zanamivir treatment for 7 days because of possible resistance to oseltamivirB. Standard-dose oseltamivir for 7 days to avoid potential adverse eventsC. High-dose oseltamivir for 5 days to limit antiviral exposureD. Weekly serial viral polymerase chain reaction (PCR) testing until there is a negative result

## Activity Evaluation

**Table:** 

1. The activity supported the learning objectives.				
Strongly Disagree				Strongly Agree
1	2	3	4	5
2. The material was organized clearly for learning to occur.				
Strongly Disagree				Strongly Agree
1	2	3	4	5
3. The content learned from this activity will impact my practice.				
Strongly Disagree				Strongly Agree
1	2	3	4	5
4. The activity was presented objectively and free of commercial bias.				
Strongly Disagree				Strongly Agree
1	2	3	4	5
